# On the Open Problem Related to Rank Equalities for the Sum of Finitely Many Idempotent Matrices and Its Applications

**DOI:** 10.1155/2014/702413

**Published:** 2014-07-10

**Authors:** Mei-xiang Chen, Qing-hua Chen, Qiao-xin Li, Zhong-peng Yang

**Affiliations:** ^1^School of Mathematics and Computer Science, Fujian Normal University, Fuzhou, Fujian 350007, China; ^2^School of Mathematics, Putian University, Putian, Fujian 351100, China; ^3^Institute of Applied Physics and Computational Mathematics, Beijing 100094, China

## Abstract

Tian and Styan have shown many rank equalities for the sum of two and three idempotent matrices and pointed out that rank equalities for the sum *P*_1_ + ⋯+*P*_*k*_ with *P*_1_,…, *P*_*k*_ be idempotent (*k* > 3) are still open. In this paper, by using block Gaussian elimination, we obtained rank equalities for the sum of finitely many idempotent matrices and then solved the open problem mentioned above. Extensions to scalar-potent matrices and some related matrices are also included.

## 1. Introduction

Let *C* ^*m*×*n*^ and *GL*_*n*_(*C*) be the sets of *m* × *n* complex matrices and *n* × *n* nonsingular matrices, respectively. The *n* × *n* identity matrix is denoted by *I*_*n*_ or simply by *I* if the size is immaterial. Let *Z* ^+^ be the set of all the positive integer numbers. The symbols *r*(*A*) and *A* ^*T*^ stand for the rank and transpose of *A* ∈ *C* ^*m*×*n*^, respectively, while tr⁡*A* denotes the trace of a square matrix *A*. A matrix *A* ∈ *C* ^*n*×*n*^ is said to be* idempotent*, if *A* ^2^ = *A*, and* scalar*-*potent* (determined by *λ*), if *A* ^2^ = *λA*, for some (0≠)*λ* ∈ *C* (see, e.g., [1]). When *λ* = 1, it coincides with the definition of an idempotent matrix.

As one of the fundamental building blocks in matrix theory, idempotent matrices are very useful in many contexts and have been extensively studied in the literature (see, e.g., [1–6]). Here we focus on the research on the rank of the sum of idempotent matrices.

Gröss and Trenkler have studied rank of the sum of two idempotent matrices (see [3, Theorem 3]). Also, Tian and Styan have shown a rank equality for two idempotent matrices as follows.

**Proposition 1 (see [1, Theorem 2.4] and [2, Theorem 2.1]).**

Let *P*, *Q* ∈ *C* ^*n*×*n*^ be idempotent. Then

(1) $$\begin{array}{c} r\left(P+Q\right)=r\left(\begin{array}{cc} P & Q\\ Q & 0 \end{array}\right)-r\left(Q\right). \end{array}$$

Tian and Styan have extended the rank equality for the sum of idempotent matrices to the scalar-potent matrices (see, e.g., [1]).

**Proposition 2 (see [1, P110]).**

Let *P*, *Q* ∈ *C* ^*n*×*n*^ be scalar-potent matrices determined by nonzero complexes *λ*, *μ*. Then

(2) r(μP+λQ)=r(PQQ0)−r(Q),P2=λP, Q2=μQ, λμ≠0.

Later, Tian and Styan considered the rank equality for the sum of three idempotent matrices in [2] as follows.

**Proposition 3 (see [2, P95]).**

Let *P*_1_, *P*_2_, *P*_3_ ∈ *C* ^*n*×*n*^ be idempotent. Then

(3) $$\begin{array}{c} r\left(P_{1}+P_{2}+P_{3}\right)=r\left(\begin{array}{ccc} \frac{1}{2}P_{1} & P_{2} & P_{3}\\ P_{2} & 0 & P_{2}P_{3}\\ P_{3} & P_{3}P_{2} & 0 \end{array}\right)-r\left(P_{2}\right)-r\left(P_{3}\right). \end{array}$$

By (3), Tian and Styan have induced many useful results, for example, if *P*_1_, *P*_2_, *P*_3_ are idempotent and *P*_1_ + *P*_2_ + *P*_3_ = 0, then *P*_1_ = *P*_2_ = *P*_3_ = 0. The literatures [2, 4–6] show that establishing various kinds of rank equalities for *k* idempotent matrices is interesting. Tian and Styan pointed out that rank equalities for the sum *P*_1_ + ⋯+*P*_*k*_ with *P*_1_,…, *P*_*k*_ be idempotent (*k* > 3) are still open (see [2, P95]).

In this paper, by applying block Gaussian elimination, rank equalities for the sum of finitely many idempotent matrices are obtained. These results generalize (3) and solve the open problem proposed by Tian and Styan (see, e.g., [2]). Also, new rank equalities for finitely many idempotent matrices are given. The rank equality (3) is generalized to *k* scalar-potent matrices as well.

## 2. Main Results

Before showing main results, we need some preparations.

**Lemma 4 .**

Let *A*_1_,…, *A*_*k*_ ∈ *C* ^*n*×*m*^. Then

(4) $$\begin{array}{c} r\left(\begin{array}{ccccc} A_{1} & 0 & \cdots & 0 & A_{1}\\ 0 & A_{2} & \cdots & 0 & A_{2}\\ \cdots & \cdots & \ddots & \cdots & \cdots \\ 0 & 0 & \cdots & A_{k} & A_{k}\\ A_{1} & A_{2} & \cdots & A_{k} & 0 \end{array}\right)=\overset{k}{\underset{i=1}{\sum }}r\left(A_{i}\right)+r\left(\overset{k}{\underset{i=1}{\sum }}A_{i}\right), \end{array}$$

for any *k* ∈ *Z* ^+^.

**Proof**

Let

(5) $$\begin{array}{c} G=\left(\begin{array}{ccccc} I_{n} & 0 & \cdots & 0 & 0\\ 0 & I_{n} & \cdots & 0 & 0\\ \cdots & \cdots & \ddots & \cdots & \cdots \\ 0 & 0 & \cdots & I_{n} & 0\\ -I_{n} & -I_{n} & \cdots & -I_{n} & I_{n} \end{array}\right)\in C^{n(k+1)\times n(k+1)},\\ S=\left(\begin{array}{ccccc} I_{m} & 0 & \cdots & 0 & -I_{m}\\ 0 & I_{m} & \cdots & 0 & -I_{m}\\ \cdots & \cdots & \ddots & \cdots & \cdots \\ 0 & 0 & \cdots & I_{m} & -I_{m}\\ 0 & 0 & \cdots & 0 & I_{m} \end{array}\right)\in C^{m(k+1)\times m(k+1)}. \end{array}$$

It is evident that *G* and *S* are nonsingular.

By calculation,

(6) G(A10⋯0A10A2⋯0A2⋯⋯⋱⋯⋯00⋯AkAkA1A2⋯Ak0)S  =diag⁡(A1,…,Ak,−∑i=1kAi);

since *G* and *S* are nonsingular, hence

(7) r(A10⋯0A10A2⋯0A2⋯⋯⋱⋯⋯00⋯AkAkA1A2⋯Ak0)  =r(diag⁡(A1,…,Ak,−∑i=1kAi))  =∑i=1kr(Ai)+r(∑i=1kAi).

This completes the proof.

The proof method of Lemma 4 is inspired by Marsaglia and Styan [5, Theorem 9]. By (4), we get the rank equality for the sum of finitely many idempotent matrices; it is different from the one of three idempotent matrices (3) given by Tian and Styan. Consequently, to find the generalization of Proposition 3 and solve the open problem given by Tian and Styan (see, e.g., [2]), it is necessary to seek a new method different from Lemma 4.

**Lemma 5 (see [7, Problem 4.9]).**

Let *P* ∈ *C* ^*n*×*n*^ be idempotent. Then *r*(*P*) = tr⁡*P*.

In this section, from now on, for *A*_1_, *A*_2_,…, *A*_*k*_ ∈ *C* ^*n*×*n*^, one denotes

(8) W(A1,A2,…,Ak)=(12A1A2A3⋯Ak−1AkA20A2A3⋯A2Ak−1A2AkA3A3A20⋯A3Ak−1A3Ak⋮⋮⋮⋱⋮⋮Ak−1Ak−1A2Ak−1A3⋯0Ak−1AkAkAkA2AkA3⋯AkAk−10)∈Cnk×nk.

**Theorem 6 .**

For any *k* ∈ *Z* ^+^, let *P*_1_,…, *P*_*k*_ ∈ *C* ^*n*×*n*^ be idempotent. Then

(9) r(∑i=1kPi)  =r(W(P1,…,Pk))−∑i=2kr(Pi)  =r(W(P1,…,Pk))−∑i=2ktr⁡Pi  =r(W(P1,…,Pk))−tr⁡(∑i=2kPi).

**Proof**

From Lemma 4, it follows that

(10) $$\begin{array}{c} r\left(\begin{array}{ccccc} P_{1} & 0 & \cdots & 0 & P_{1}\\ 0 & P_{2} & \cdots & 0 & P_{2}\\ \cdots & \cdots & \ddots & \cdots & \cdots \\ 0 & 0 & \cdots & P_{k} & P_{k}\\ P_{1} & P_{2} & \cdots & P_{k} & 0 \end{array}\right)=\overset{k}{\underset{i=1}{\sum }}r\left(P_{i}\right)+r\left(\overset{k}{\underset{i=1}{\sum }}P_{i}\right). \end{array}$$

On the other hand, by block Gaussian elimination, we will see that

(11) $$\begin{array}{c} r\left(\begin{array}{ccccc} P_{1} & 0 & \cdots & 0 & P_{1}\\ 0 & P_{2} & \cdots & 0 & P_{2}\\ \cdots & \cdots & \ddots & \cdots & \cdots \\ 0 & 0 & \cdots & P_{k} & P_{k}\\ P_{1} & P_{2} & \cdots & P_{k} & 0 \end{array}\right)=r\left(W\left(P_{1},\ldots ,P_{k}\right)\right)+r\left(P_{1}\right). \end{array}$$

In fact, let us write the matrix

(12) $$\begin{array}{c} \left(\begin{array}{ccccc} P_{1} & 0 & \cdots & 0 & P_{1}\\ 0 & P_{2} & \cdots & 0 & P_{2}\\ \cdots & \cdots & \ddots & \cdots & \cdots \\ 0 & 0 & \cdots & P_{k} & P_{k}\\ P_{1} & P_{2} & \cdots & P_{k} & 0 \end{array}\right) \end{array}$$

as the quadripartitioned matrix

(13) $$\begin{array}{c} \left(\begin{array}{cc} M_{11} & M_{12}\\ M_{21} & 0 \end{array}\right)\in C^{n(k+1)\times n(k+1)}, \end{array}$$

where

(14) M11=diag⁡(P1,…,Pk)∈Cnk×nk,  M12=(P1T,…,PkT)T∈Cnk×n,M21=(P1,…,Pk)∈Cn×nk.

By (11) and (13), it suffices to show

(15) $$\begin{array}{c} r\left(\begin{array}{cc} M_{11} & M_{12}\\ M_{21} & 0 \end{array}\right)=r\left(W\left(P_{1},\ldots ,P_{k}\right)\right)+r\left(P_{1}\right). \end{array}$$

Direct calculations to (13) show that

(16) $$\begin{array}{c} \left(\begin{array}{cc} I_{nk} & W_{12}\\ 0 & I_{n} \end{array}\right)\left(\begin{array}{cc} M_{11} & M_{12}\\ M_{21} & 0 \end{array}\right)=\left(\begin{array}{cc} M_{11}+W_{12}M_{21} & M_{12}\\ M_{21} & 0 \end{array}\right), \end{array}$$

where

(17) $$\begin{array}{c} W_{12}={\left(0,-P_{2}^{T},\ldots ,-P_{k}^{T}\right)}^{T}\in C^{nk\times n},\\ \left(\begin{array}{cc} I_{nk} & W_{12}\\ 0 & I_{n} \end{array}\right)\in GL_{n(k+1)}\left(C\right). \end{array}$$

If we define *X*_11_ = *M*_11_ + *W*_12_ *M*_21_, by (14) and (17), we get

(18) $$\begin{array}{c} X_{11}=\left(\begin{array}{ccccc} P_{1} & 0 & \cdots & 0 & 0\\ -P_{2}P_{1} & 0 & \cdots & -P_{2}P_{k-1} & -P_{2}P_{k}\\ \cdots & \cdots & \ddots & \cdots & \cdots \\ -P_{k-1}P_{1} & -P_{k-1}P_{2} & \cdots & 0 & -P_{k-1}P_{k}\\ -P_{k}P_{1} & -P_{k}P_{2} & \cdots & -P_{k}P_{k-1} & 0 \end{array}\right). \end{array}$$

Moreover, let

(19) $$\begin{array}{c} S_{21}=\left(-\frac{1}{2}E_{n},0,\ldots ,0\right),\;\;G_{21}=\left(P_{1},0,\ldots ,0\right),\;\;\;\\ R_{12}={\left(-\frac{1}{2}P_{1}^{T},0,\ldots ,0\right)}^{T}; \end{array}$$

then

(20) $$\begin{array}{c} \left(\begin{array}{cc} I_{nk} & 0\\ S_{21} & I_{n} \end{array}\right),\left(\begin{array}{cc} I_{nk} & 0\\ G_{21} & I_{n} \end{array}\right),\left(\begin{array}{cc} I_{nk} & R_{12}\\ 0 & I_{n} \end{array}\right)\;\;\text{are}\;\;\text{nonsingular}. \end{array}$$

By (14) and (19), we see that

(21) X11+M12G21  =(2P10⋯0000⋯−P2Pk−1−P2Pk⋯⋯⋯⋯⋯0−Pk−1P2⋯0−Pk−1Pk0−PkP2⋯−PkPk−10)  ∈Cnk×nk.

Then by applying (14) and (19) yields

(22) $$\begin{array}{c} \left(X_{11}+M_{12}G_{21}\right)R_{12}+M_{12}={\left(0,P_{2}^{T},\ldots ,P_{k}^{T}\right)}^{T}\in C^{nk\times n}. \end{array}$$

Thus,

(23) S21X11+M21+S21M12G21 =S21(X11+M12G21)+M21 =(−12I,0,…,0)  ×(2P10⋯0000⋯−P2Pk−1−P2Pk⋯⋯⋯⋯⋯0−Pk−1P2⋯0−Pk−1Pk0−PkP2⋯−PkPk−10)  +(P1,P2,…,Pk) =(0,P2,…,Pk).

Hence it follows from (14) and (19) that

(24) $$\begin{array}{c} \left(S_{21}X_{11}+M_{21}+S_{21}M_{12}G_{21}\right)R_{12}+S_{21}M_{12}=-\frac{1}{2}P_{1}. \end{array}$$

Consequently, from (16)–(24), it follows that

(25) (Ink0S21In)(InkW120In)(M11M12M210)(Ink0G21In)(InkR120In)  =(Ink0S21In)(X11M12M210)(Ink0G21In)(InkR120In)  =(X11+M12G21(X11+M12G21)R12+M12S21X11+M21+S21M12G21(S21X11+M21+S21M12G21)R12+S21M12)  =(2P10⋯00000⋯−P2Pk−1−P2PkP2⋯⋯⋱⋯⋯0−Pk−1P2⋯0−Pk−1PkPk−10−PkP2⋯−PkPk−10Pk0P2⋯Pk−1Pk−12P)  =(2P100(0⋯−P2Pk−1−P2PkP2⋯⋱⋯⋯−Pk−1P2⋯0−Pk−1PkPk−1−PkP2⋯−PkPk−10PkP2⋯Pk−1Pk−12P))  =diag⁡(2P1,Z),

where

(26) Z=((0⋯−P2Pk−1−P2Pk⋯⋯⋯⋯−Pk−1P2⋯0−Pk−1Pk−PkP2⋯−PkPk−10)(P2⋮Pk−1Pk)(P2,…,Pk−1,Pk)−12P1)=(Z11Z12Z21−12P1)∈Cnk×nk.

Since $\left(\begin{array}{cc} I_{nk} & 0\\ s_{21} & I_{n} \end{array}\right)$, $\left(\begin{array}{cc} I_{nk} & W_{12}\\ 0 & I_{n} \end{array}\right)$, $\left(\begin{array}{cc} I_{nk} & 0\\ G_{21} & I_{n} \end{array}\right)$ and $\left(\begin{array}{cc} I_{nk} & R_{12}\\ 0 & I_{n} \end{array}\right)$ are nonsingular, we get

(27) $$\begin{array}{c} r\left(\begin{array}{cc} M_{11} & M_{12}\\ M_{21} & 0 \end{array}\right)=r\left(P_{1}\right)+r\left(\begin{array}{cc} Z_{11} & Z_{12}\\ Z_{21} & -\frac{1}{2}P_{1} \end{array}\right). \end{array}$$

Also, it is easy to verify that

(28) (0In−Ink0)(Z11Z12Z21−12P1)(0Ink−In0)  =(12P1Z21Z12−Z11)=W(P1,…,Pk).

Since $\left(\begin{array}{cc} 0\; & I_{n}\\ -I_{nk} & 0 \end{array}\right)$ and $\left(\begin{array}{cc} 0 & I_{nk}\\ -I_{n} & 0 \end{array}\right)$ are nonsingular, by (13) and (26)–(28), we obtain

(29) r(M11M12M210)=r(P1)+r(Z)=r(P1)+r(W(P1,…,Pk)).

Combining (13) with (29) together with Lemma 5 yields the desired results.

When *k* = 3, Theorem 6 leads to Proposition 3 at once, and when *k* = 2, it leads to Proposition 1; for the idempotent matrices *P* and *Q*, it follows that

(30) (2In00In)W(P,Q)(In0012In)  =(2In00In)(12PQQ0)(In0012In)=(PQQ0).

For the sum of two idempotent matrices, Tian and Styan have given out many rank equalities (see [1, Theorem 2.4] and [2, Theorems 2.1, 2.3, 2.4, and 2.7]). Let *P*, *P*_1_,…, *P*_*k*_ ∈ *C* ^*n*×*n*^ be idempotent; using Theorem 6 together with [6, Theorem 6] and [6, (26)] yields the equalities as follows:

(31) r(∑i=1kPi) =r(W(P1,…,Pk))+r(P1)  +r(P1−P2P1−P3⋮P1−Pk)−r(P1P2⋮Pk)−r(P1P2P1P3⋮⋱P1Pk),

(32) r(P+∑i=1kPi)=r(W(P,P1,…,Pk))+r(P). +r(P−kP1P⋯PPP−kP2⋯P⋮⋮⋱⋮PP⋯P−kPk) −r(PP⋯PP1P2…Pk)−r(PP1PP2⋮⋱PPk).

Theorem 6 together with (31) and (32) indicates that the sum of *k*(≥3) idempotent matrices has various kinds of rank equalities, as shown in the discussions in the literature [1, 2].

In view of (11), by applying Lemma 5, we see the following.

**Corollary 7 .**

For any *k* ∈ *Z* ^+^, let *P*_1_,…, *P*_*k*_ ∈ *C* ^*n*×*n*^ be idempotent. Then

(33) r(P10⋯0P10P2⋯0P2⋯⋯⋯⋯⋯00⋯PkPkP1P2⋯Pk0)−r(W(P1,…,Pk))  =r(P1)=tr⁡P1.

This immediately implies that the difference of the ranks of two block matrices in the left side of (33) is always equal to *r*(*P*_1_) or tr⁡*P*_1_, independently on the choice of *k*, when *k* ≥ 3.

## 3. The Rank Formulas for the Sum of *k* Scalar-Potent Matrices and Applications

Theorem 6 can easily be extended to scalar-potent matrices; in fact,

(34) (1λiPi)2=1λiPi,  r(1λiPi)=r(Pi),Pi2=λiPi, i=1,…,k.

So (1/*λ*_*i*_)*P*_*i*_ is idempotent.

**Theorem 8 .**

For any given *k* ∈ *Z* ^+^, let *P*_*i*_ ∈ *C* ^*n*×*n*^ be scalar-potent (determined by *λ*_*i*_(≠0)), *i* = 1,…, *k*. Then

(35) r[∑i=1k(∏j≠iλj)Pi]  =r(W(1λ1P1,P2,…,Pk))−∑i=2kr(Pi)  =r(W(1λ1P1,P2,…,Pk))−∑i=2k1λitr⁡Pi.

**Proof**

By (8) and (34), we get

(36) W(1λ1P1,…,1λkPk)  =(12λ1P11λ2P21λ3P3⋯1λk−1Pk−11λkPk1λ2P201λ2λ3P2P3⋯1λ2λk−1P2Pk−11λ2λkP2Pk1λ3P31λ3λ2P3P20⋯1λ3λk−1P3Pk−11λ3λkP3Pk⋮⋮⋮⋱⋮⋮1λk−1Pk−11λk−1λ2Pk−1P21λk−1λ3Pk−1P3⋯01λk−1λkPk−1Pk1λkPk1λkλ2PkP21λkλ3PkP3⋯1λkλk−1PkPk−10).

On the other hand, using (36), we obtain

(37) $$\begin{array}{c} W\left(\frac{1}{\lambda_{1}}P_{1},P_{2},\ldots ,P_{k}\right)=GW\left(\frac{1}{\lambda_{1}}P_{1},\ldots ,\frac{1}{\lambda_{k}}P_{k}\right)G, \end{array}$$

with *G* = diag⁡(*I*_*n*_, *λ*_2_ *I*_2_,…, *λ*_*k*_ *I*_*n*_). Since *G* is nonsingular, by (37), we can write

(38) r(W(1λ1P1,P2,…,Pk))  =r(W(1λ1P1,1λ2P2,…,1λkPk)).

From (34), (1/*λ*_*i*_)*P*_*i*_ is idempotent. Using Theorem 6 together with (38) yields the equality

(39) r(∑i=1k1λiPi)  =r(W(1λ1P1,1λ2P2,…,1λkPk))−∑i=2kr(1λiPi)  =r(W(1λ1P1,P2,…,Pk))−∑i=2kr(1λiPi).

We note that

(40) r(∑i=1k1λiPi)=r[(∏j=1kλj)(∑i=1k1λiPi)]=r[∑i=1k(∏j≠iλj)Pi].

From (39) and (40), we get the desired result since *r*(*P*_*i*_) = *r*((1/*λ*_*i*_)*P*_*i*_) = (1/*λ*_*i*_)tr⁡*P*_*i*_.

When *k* = 2, this leads immediately to Proposition 2, since it can be written as

(41) $$\begin{array}{c} \left(\begin{array}{cc} 2\lambda I_{n} & 0\\ 0 & I_{n} \end{array}\right)\left(\begin{array}{cc} \frac{1}{2\lambda }P & Q\\ Q & 0 \end{array}\right)\left(\begin{array}{cc} I_{n} & 0\\ 0 & \frac{1}{2\lambda }I_{n} \end{array}\right)=\left(\begin{array}{cc} P & Q\\ Q & 0 \end{array}\right), \end{array}$$

with *P* ^2^ = *λP*,  *Q* ^2^ = *μQ*, and  *λμ* ≠ 0.

For any given idempotent matrix *P*, Farebrother and Trenkler [8] denoted the set of generalized quadratic matrices as

(42) Ωn(P)={A∈Cn×n:A2=αA+βP,AP=PA=A, ∀α,β∈C}.

If *P* = *I*, it coincides with the definition of a quadratic matrix (see, e.g., [9]). In view of [10, Lemma 1] and [11, Lemma 2.2], we conclude that (42) can be expressed equivalently as

(43) Ωn(P) ={A∈Cn×n:(A−aP)(A−bP)=0,   AP=PA=A,∀a,b∈C}.

If *A* ∈ Ω_*n*_(*P*), then from (42) and (43), we see that

(44) $$\begin{array}{c} a=\frac{1}{2}\left(\alpha +\sqrt{\alpha^{2}+4\beta }\right),\\ b=\frac{1}{2}\left(\alpha -\sqrt{\alpha^{2}+4\beta }\right). \end{array}$$

**Lemma 9 .**

For any given idempotent matrix *P*, if *A* ∈ Ω_*n*_(*P*) satisfies (*A* − *aP*)(*A* − *bP*) = 0 with *a* ≠ *b*, then *A* − *aP* is a scalar-potent matrix determined by *b* − *a*.

**Proof**

For the matrix *P*, there exists a nonsingular matrix *S* such that *P* = *S*diag⁡(*I*_*k*_, 0)*S* ^−1^. From *AP* = *PA*, we can write *A* = *S*diag⁡(*A*_1_, *A*_2_)*S* ^−1^ being *A*_1_ ∈ *C* ^*k*×*k*^. From *AP* = *A*, we get *A*_2_ = 0; namely, *A* = *S*diag⁡(*A*_1_, 0)*S* ^−1^. We have (*A*_1_ − *aI*_*k*_)(*A*_1_ − *bI*_*k*_) = 0. It is seen from the fact that a matrix is diagonalizable if and only if its minimal polynomial has simple roots (see [12, Corollary 3.3.10]). Thus, there exists a nonsingular matrix *S*_1_ such that *A*_1_ = *S*_1_diag⁡(*aI*_*r*_, *bI*_*k*−*r*_)*S*_1_^−1^. Let *R* = diag⁡(*S*_1_, *I*); then *R* is nonsingular and

(45) $$\begin{array}{c} P=R\mathrm{diag}\left(I_{r},I_{k-r},0\right)R^{-1},\\ A=R\mathrm{diag}\left(aI_{r},bI_{k-r},0\right)R^{-1}. \end{array}$$

Hence

(46) A−aP=Rdiag⁡(0,(b−a)Ik−r,0)R−1=(b−a)Rdiag⁡(0,In−r,0)R−1.

Now, it is evident that (*A* − *aP*)^2^ = (*b* − *a*)(*A* − *aP*).

**Theorem 10 .**

For any given idempotent matrices *P*_1_,…, *P*_*k*_ ∈ *C* ^*n*×*n*^ and any *k* ∈ *Z* ^+^, if *A*_*i*_ ∈ Ω_*n*_(*P*_*i*_) satisfies (*A*_*i*_ − *a*_*i*_ *P*_*i*_)(*A*_*i*_ − *b*_*i*_ *P*_*i*_) = 0 with *a*_*i*_ ≠ *b*_*i*_, *i* = 1,…, *k*, then

(47) r[∑i=1k(∏j≠i(bj−aj))(Ai−aiPi)]=r(W(1b1−a1(A1−a1P1),A2−a2P2,…,Ak−akPk)) −∑i=2kr(Ai−aiPi)=r(W(1b1−a1(A1−a1P1),A2−a2P2,…,Ak−akPk)) −∑i=2k1bi−aitr⁡(Ai−aiPi).

**Proof**

For the idempotent matrices *P*_*i*_, by applying Lemma 9, we see that *A*_*i*_ − *a*_*i*_ *P*_*i*_ is a scalar-potent matrix determined by *b*_*i*_ − *a*_*i*_; then results follow from Theorem 8.
